# Supplementary material for: Regularized sequence-context mutational trees capture variation in mutation rates across the human genome
Source: PLoS Genet. 2023 Jul 7;19(7):e1010807. doi: 10.1371/journal.pgen.1010807 (PMC10355397; doi:10.1371/journal.pgen.1010807)
Supplement: S3 Text — (DOCX) [file pgen.1010807.s018.docx]

# **S3 Text – Data Availability**

We have implemented our Baymer method into software that is freely available as a python package. This can be accessed on the Voight Lab GitHub repository: <https://github.com/bvoightlab/Baymer/>. Additional outputs generated by the model presented in this work are also available at: <https://doi.org/10.5281/zenodo.7843023>. All data analyzed here are publicly available at the following websites:

**NYGC resequencing of 1KG Phase III data**[1]**:**

http://ftp.1000genomes.ebi.ac.uk/vol1/ftp/data_collections/1000G_2504_high_coverage/working/20190425_NYGC_GATK/

**gnomADv3.0**[2]**:**

https://gnomad.broadinstitute.org/downloads

**Halldorsson et al. trio data**[3]**:**

https://science.sciencemag.org/highwire/filestream/721792/field_highwire_adjunct_files/7/aau1043_DataS5_revision1.tsv

**1KG accessibility mask**[4]**:**

http://ftp.1000genomes.ebi.ac.uk/vol1/ftp/data_collections/1000_genomes_project/working/20160622_genome_mask_GRCh38/PilotMask/20160622.allChr.pilot_mask.bed

**RefSeq coding regions**[5]**:**

http://www.ensembl.org/biomart/

**Ancestral FASTA**[6]**:**

<ftp://ftp.ensembl.org/pub/release97/fasta/ancestral_alleles/homo_sapiens_ancestor_GRCh38.tar.gz>

**Great Ape Genome Project Data**[7]**:**

https://www.biologiaevolutiva.org/greatape/data.html

**References**

1. Byrska-Bishop M, Evani US, Zhao X, Basile AO, Abel HJ, Regier AA, et al. High-coverage whole-genome sequencing of the expanded 1000 Genomes Project cohort including 602 trios. Cell. 2022;185(18):3426–40.

2. Lek M, Karczewski KJ, Minikel E V., Samocha KE, Banks E, Fennell T, et al. Analysis of protein-coding genetic variation in 60,706 humans. Nature. 2016;

3. Halldorsson B V, Palsson G, Stefansson OA, Jonsson H, Hardarson MT, Eggertsson HP, et al. Characterizing mutagenic effects of recombination through a sequence-level genetic map. Science (1979). 2019;363(6425):eaau1043.

4. 1000 Genomes Project Consortium, Auton A, Brooks LD, Durbin RM, Garrison EP, Kang HM, et al. A global reference for human genetic variation. Nature. 2015;

5. O’Leary NA, Wright MW, Brister JR, Ciufo S, Haddad D, McVeigh R, et al. Reference sequence (RefSeq) database at NCBI: current status, taxonomic expansion, and functional annotation. Nucleic Acids Res. 2016;44(D1):D733–45.

6. Ensembl. Ensembl, Data from “homo_sapiens_ancestor_GRCh38.” http://ftp.ensembl.org/pub/release-97/fasta/ancestral_alleles/homo_sapiens_ancestor_GRCh38.tar.gz.

7. Prado-Martinez J, Sudmant PH, Kidd JM, Li H, Kelley JL, Lorente-Galdos B, et al. Great ape genetic diversity and population history. Nature [Internet]. 2013;499(7459):471–5. Available from: https://doi.org/10.1038/nature12228
