# Supplementary material for: Regularized sequence-context mutational trees capture variation in mutation rates across the human genome
Source: PLoS Genet. 2023 Jul 7;19(7):e1010807. doi: 10.1371/journal.pgen.1010807 (PMC10355397; doi:10.1371/journal.pgen.1010807)
Supplement: Attachment — Submitted filename: baymer_rebuttal_FINAL.docx [file pgen.1010807.s019.docx]

**We sincerely thank the reviewers and editors for their careful reading of the manuscript and their criticisms and suggestions. We have taken their feedback to heart in this revision, especially in improving our model descriptions and algorithmic decision-making, which we think has substantially improved this revised manuscript. Reviewer’s comments are in *italics*, and our specific responses are below in bold.**

*# Reviewer 1*

*In this manuscript, Adams et al describe a method to model the likelihood of mutations occuring in the human germline given the (up to) 9-mer nucleotide context of the mutated locus. The method incorporates a form of regularization (via the spike-and-slab prior) helping it deal with sparse/noisy data due to low mutation counts and thus lessens overfitting, and further provides estimates of uncertainty. The hierarchical nature of the model allows for a certain form of transfer-learning-like strategy. The modelling method is well described, and reasonably rigorously tested on out-of-sample data and it is convincing that it can model the extended, up to nonanucleotide, context effects on mutation rates in the human germline well.*

**Author Response: We thank the reviewer for their careful reading and positive feedback regarding the model description and evaluation.**

*With respect to novel biological insight gained, this study is in my opinion somewhat limited. All the analysis is on human germline mutation/polymorphism. We see that there is signal in the 9-mer context in human population variation, extended from the 7-mer reported in a previous paper from the same lab (DOI: 10.1038/ng.3511); this is arguably a bit of an incremental advance. There are reports about differences („shifts“) in mutable oligonucleotides between human populations, with again largely unclear novelty compared to another previous paper from the same lab (doi: 10.1093/molbev/msz023). In all fairness, here, because of the new method, we do have the ability to check the variation in the 9mer contexts (wider than before) however significant differences appear very rare (or, with very slight effect, so they 'slip under the radar').*

**Author Response: We appreciate the author’s point here. To be clear, we believe that our methodological contributions include:**

a. **Incorporation of uncertainty plus regularization into the estimation procedure. This is also important for purposes of parsimony – when evaluating smaller sized data sets or smaller genomic territory, data will be less abundant, and thus a tool that models as far as data allows and passes uncertainty is especially crucial. Reasonably minds might reasonably disagree, but we believe that capturing uncertainty is a non-trivial contribution, particularly if one is interested in performing any types of comparison, especially in circumstances where genomic territory is limited. Our own previous work carried this as a limitation.**
b. **Evaluation and demonstration that windows of 9-mer context explain patterns of variation better than previous sized windows of context, more than our previous report (focused on hepta-nucleotide contexts).**

**In addition, we have added a range of additional analyses to the revision, which we hope further extends interest and expands the range of possible applications which we discuss below.**

*There are some technical tests on what sort of polymorphisms (singletons, doubletons, rare variants, species divergence) represent the best proxy for de novo mutations, which is interesting as an approach to boost the accuracy of the methodology, but it is not clear what new biology is/can be revealed using the improvements. In this study, the method is applied in a specialized way, only to the population variants and de novo variants, without demonstrating more general application beyond population genomics. This would be shown by illustrative analyses of feasibility to apply to other data types, e.g. somatic mutations, species divergence (except as a baseline, which was implemented), or population variation in other species). Or, as touched upon in the discussion, application to different genomic regions („areas“) would be of much interest, since it can add very interesting context to germline variation analysis (e.g. DNA replication time associates with mutagenesis quite strongly, and is often considered in genome-wide mutagenesis analyses). Thus the paper also misses, in my opinion, showcasing of the method on more diverse set of applications, to demonstrate broad interest.*

**Author Response: We appreciate what the reviewer desires here. Based on this and additional comments from reviewers, in the revised manuscript, we have included the following additional experiments which bring forward additional novelty:**

a. **The demonstration that asymmetric models do in fact provide for better fit models with far fewer parameters than symmetric ones. We have created an additional section in results and have included an entire new display item (Figure 2) describing that result.**
b. **We have included analysis suggested by this Reviewer to explore differences in estimated rates across the site frequency spectrum, noting little if any major differences there (see Reviewer #1, comment #2)**
c. **We have brought forward examination of sequencing contexts with the largest probability changes across layers associated with them (Supplementary Table 3). These include well known effects (e.g., CpGs), those we have previously reported (e.g., elevated A>G rates for the CAAT motif, lower rates for certain poly-nucleotide tracts), but some new effects at higher order context (8-mer, 9-mer). For each mutation type, we also look closely at sequence context for the top 100 largest shifts, identifying additional sub-motifs in the 9-mer window that appear to be correlated with the top and bottom 1% of polymorphism probabilities.**
d. **Finally, to further showcase the model, we took advantage of extant polymorphism data from non-human, great ape species, comparing rates between those species and humans. We note conservation broadly across context-inferred rate variability shifting somewhat proportional to estimated divergence time. We demonstrate that human models can perform well as proxies of other great ape species.**

**We believe these additions constitute improvements in how to capture rate variation and an approach that can help dissect investigators to further explore rate differences and correlates of those rates with features across the genome and across species.**

*Major comments*

*1. Lines 182-184, describing the concordance test: "If zero-mutation contexts omitted from the maximum likelihood model were included, the correlations would perform considerably worse (Methods, Supplementary Fig. 1D, ρ = 0.876; RMSPE = 0.744)". Could the authors explain why two clouds of points appear at the top left and bottom right of the plot? Are these variants with exactly zero counts?*

**Author Response: We agree and the reviewer’s intuition is correct. These are contexts which have no mutations in one of the two datasets, and thus frequentist estimates are driven exclusively by pseudo-counts. We have made more explicit note of this in the manuscript, both in the main text (lines 230-234 of the Main Text) and in the Supplementary Figure 1 caption.**

*2. Additionally, it may be interesting to consider correlation in the 9-mer model by considering bins of common variants with larger counts (for instance, allele frequency >5%, >10%, 20% etc) – is Baymer expected to perform similarly well on variants under selection? (I expect that it should; they may want to comment and/or show with data)*

**Author Response:** **We agree with the reviewer. For example, in our previous work (Aggarwala and Voight 2016) with codon-aware expanded sequence context models that use the noncoding genome as a prior captured variability better than simple codon or trinucleotide models in exons. Based on this, we have no reason to believe that a Baymer model would behave differently in regions which are subject to constraint. Furthermore, it is worth remembering that our analyses focus on the subset of the non-coding genome where we think the effects of background selection are the most minimal.**

**However, to fully test the most obviously constrained region of the genome – the exome – we would need to create a heavily modified implementation of Baymer that was sensitive to and integrated information across codon models - not an immediately obvious extension to us when considering it. We do hope the reviewer could appreciate that this type of direction is now well motivated by results presented in this revised manuscript. Owing to this generally important point, we have added a comment on this in the discussion (lines 531-536 in Main Text).**

**The previous version did not measure consistency between allele frequency bins, as suggested by the reviewer. We agree with them of their value. We have performed experiments across a variety of AF bins and note broad agreement after adjusting for total mutation counts and added that to the manuscript in the results (lines 233-234 in the Main Text).**

*3. Based on the power analysis presented in Table 2 (which is well appreciated!), which provides the rate of false-negatives at various effect sizes, could the authors infer the full number of true shifts in the various the 3-mer, 5-mer, 7-mer, 9-mer contexts? Can this be inferred from the number of discovered shifts (in true populations), and the false negative rate (estimated from analysis of simulated populations)?*

**Author Response: We thank the reviewer for this thought. Early on, we had considered this sort of approach (i.e., analogous to previous types of experiments performed in the complex-trait genomic space, to estimate genetic architecture - effect size x allele frequency - based on observed distribution of GWAS discoveries and the power to discover them).**

**However, there are two major concerns with this line of inquiry. First, the sparsity of discovered differences that we do observe, combined with the scale to which power erodes as a function of weak (subtle) effects of mutation that probably exist, we felt this analysis would be very challenging to interpret. Ultimately, we do not think right now we have a sufficient number of “positive” examples to robustly estimate architecture with reasonable levels of uncertainty. Second, our analysis does not consider the effects of time vs. change in rates. Previous work has indicated that additional effect might be visible when considering this (see Gao et al. 2023; PMID: 36779395). As a result, even if we were to embark on this line of inquiry, any architectural claims we might make could easily be falsified by considering other (reasonable) models.**

**Thus, to be conservative, we prefer to retain the presentation where we report models we can confidently exclude as plausible given our specific analytical design, rather than use the data to positively estimate what could be ‘missing’ given what we observed and the power this experiment had to discover it. The presentation here at least provides a quantitative template whereby future models and inference could be assessed, which we also think has community value for such work.**

*4. Regarding the spike-and-slab prior used for regularization – its utility in preventing overfitting was nicely demonstrated. However if I understand correctly it does have at least one metaparameter (the width of the spike) and so models may benefit from optimizing this metaparameter. This is standard practice for regularized regression, usually aiming to maximize crossvalidation accuracy.*

**Author Response: The reviewer is correct that the spike-and-slab prior does have meta-parameters – the width/variance of both the spike and the slab. In the initial design of the model, we had intended to learn these parameters from the data but found convergence issues with the MCMC under this implementation, presumably due to difficulty with chain identifiability. We felt the trade-off between computation time and (marginal) performance gain was not optimal.**

**Instead, following the recommendations from the initial spike-and-slab regularization paper (George and McCulloch 1997), we decided to set a fixed value for this parameter informed by prior intuition of what a threshold of “practical significance” might be for this model. Criteria used were (i) a ratio of variances that were within the suggested bounds to avoid computational issues, (ii) an intersection of distributions such that shifts within 10% change have higher densities in the spike, and (iii) a slab distribution that was appropriately dispersed to accommodate large shifts. We agree cross-validation could further optimize our choices, however, concerned with computational complexity given the large search space and the dual optimization of both model likelihood and shift discoverability, we implemented and tested the method with a single prior. However, these parameters are fully adjustable by the user in our implementation and could be the subject of future work.**

*Minor:*

*5. cells of the last column (Population Specificity) in Table 1/supplementary Table 3 could add an asterisk in the cell indicating that this shift was or was not reported in prior work.*

**Author Response: Thank to the reviewer for this suggest and agree this would add valuable context to our findings. We have added this denotation.**

*6. line 469: ” In all of our experiments, we focused on the entirety of the accessible, non-coding genome.: I think here "accessible" is intended to mean "accessible to sequencing", which might be confused with the meaning of "chromatin accessibility" (e.g. DHS or ATAC or such)*

**Author Response: We agree with the reviewer and apologize for the confusion. This is correct. We have made clarifying adjustments in the manuscript (Line 592-593 of Main Text)**

*7. line 491: “We also compiled all sites where the high-confidence ancestral state and GRCh38 reference genome disagree, treating this collection as a call-set of derived variants.” Can they comment on whether this definition may be biased by the fact that the GRCh38 reference allele is sometimes the minor allele and not necessarily representative of the population at large?*

**Author Response: We agree with the reviewer that this is certainly possible. However, so long as the sequence contexts of such loci are randomly distributed, we do not anticipate biases in estimates, but instead would add noise to our estimates. Due to the history of the human genome project, we hypothesize that if the local sequence context at these sites is not uniformly distributed, it will be slightly shifted to more closely approximate European haplotypes, aiding the resulting model in predicting de novo variants of European origin. As more diverse representations of the genome are created, refinements could easily be made here in the future. We have added this point in the discussion (Line 553-555).**

\# Reviewer 2

*The authors propose a new Bayesian framework for estimating the probability of observing a mutation at a site as a function of the sequence context. Their method is based on using a spike and slab prior to induce sparsity combined with a hierarchical scheme to sequentially add context to the model. They apply the method to compare polymorphism data between populations and also use it in the analysis of de novo mutations. They also conduct simulations to check that the proportional*

*The method is interesting and marks a significant advance in an area that has recently seen a burst of renewed interest. The paper is well-written although there are some technical details that I feel should be clarified. PLOSGenetics is an appropriate venue for this manuscript, although I think the readers of PLOS Genetics would appreciate some additional biological interpretation (the manuscript is very focused on the methodological advance).*

**Author Response: We thank the reviewer for their careful reading and positive sentiments about the previous version (and note that some of this reviewer’s comment was cut off in the first paragraph, presumably unintentionally!). We also appreciate their comments about the appropriateness of the work for PLoS Genetics.**

**To address some points regarding biological interpretation and inference, we have included some additional experiments to this revision that we hope will further expand interest:**

a. **The demonstration that asymmetric models do in fact provide for better fit models with far fewer parameters than symmetric ones. We have created an additional section in results and have included an entire new display item (Figure 2) describing that result (see Reviewer #2, comment #1)**
b. **We have included analysis suggested by this Reviewer to explore differences in estimated rates across the site frequency spectrum, noting little if any major differences there (see Reviewer #1, comment #2)**
c. **We have brought forward examination of sequencing contexts with the largest probability changes across layers associated with them (Supplementary Table 3). These include well known effects (e.g., CpGs), those we have previously reported (e.g., elevated A>G rates for the CAAT motif, lower rates for certain poly-nucleotide tracts), but some new effects at higher order context (8-mer, 9-mer). For each mutation type, we also look closely at sequence context for the top 100 largest shifts, identifying additional sub-motifs in the 9-mer window that appear to be correlated with the top and bottom 1% of polymorphism probabilities.**
d. **Finally, to further showcase the model, we took advantage of extant polymorphism data from non-human, great ape species, comparing rates between those species and humans. We note conservation broadly across context-inferred rate variability shifting somewhat proportional to estimated divergence time. We demonstrate that human models can perform well as proxies of other great ape species.**

**We believe these additions constitute improvements in how to capture rate variation and an approach that can help dissect investigators further explore rate differences and correlates of those rates with features across the genome and across species.**

*I have two top-level concerns about the manuscript:*

*(1) Although the hierarchical scheme for adding context-dependence to the model makes a lot of sense from a statistical perspective, it seems quite odd from a more biological or mechanistic perspective. The model works by alternating between expanding the context at the 3' end and at the 5' end, and only allows extension to include more context if the last extension was informative. But this is quite odd physically because it means that whether context at the +3 position relative to the mutation can be included depends on whether the context at the -3 position was relevant. It would make much more sense for the inclusion of the +3 position to depend on whether the adjacent +2 position was relevant. E.g. this would make sense under the hypothesis that the effects on mutation rate are mediated by stretches of adjacent bases but that these stretches need not necessarily be exactly centered on the mutated position.*

**Author Response: We certainly agree with the reviewer of the less than optimal nature of this step-wise approach and is a bit awkward, from a spatial perspective.**

**While context expansions are conditional on estimates of the parent context’s polymorphism probabilities, the model does allow for intermediate nodes to be assigned to the spike without requiring all downstream nodes to be in the spike too. For example, adding an “A” to the “*CA” context might not affect polymorphism probabilities (ACA), however, the next expansion (e.g. adding an “A” 3’ -> ACAA) might have a substantial effect on the resulting probabilities and be assigned to the slab. Furthermore, additional nucleotides are always added alternating between the 5’ and 3’ ends, regardless of the degree of regularization for the shift leading to the node in question. The reviewer’s intuition is spot-on however, that if a shift is regularized due to sparsity, all subsequent shifts stemming from this context are likely to be regularized as well, since the data is nested.**

**We take this on formally in the revision, see our response below.**

*I understand that the authors have already done a great deal of work under their current scheme. But at least a spot test of e.g. expanding via a more spatially sensible hierarchy or via a symmetric widening scheme would help alleviate worries that the current scheme is leaving out important context.*

**Author Response: We agree and now include a direct investigation of this point. To formally demonstrate the value of considering asymmetric steps, we hypothesized that these ‘even’ (asymmetric) steps, regardless, would generate more likely, more parsimonious models compared to only “odd” (symmetric) step model incorporation (Figure 2A). In the revised manuscript, we include new analysis to demonstrate that:**

a. **Selecting either “left” or “right” directions generate strongly correlated rates that are ultimately emitted from the model, indicating that the rates ultimately emitted from either choice of direction are robustly inferred (Figure 2B, 2C).**
b. **That the number of parameters utilized by the model is substantially less for those “asymmetric” even-window models, compare to the symmetric, odd-window sizes (Figure 2D).**
c. **That the likelihood of the models generated by including even-sized windows with “Left” steps or even-sized windows with “Right” steps are equivalent – or in the case of 9-mers, more likely – than an alternative that only takes “symmetric” odd-sized windows (Figure 2E).**

**Taken together, despite the limitations of their formulation, the models themselves are demonstrably better fit to the observed data.**

*(2) I think the broad readership of PLOS Genetics would be interested to hear more about the biological characteristics of the fit models and what they tell us qualitatively about the nature and extent of context dependence in human mutational dynamics. For example, if you look at a random polymorphic site, what is the distribution of the size of the context that was relevant? And what is the answer for a random (not necessarily polymorphic) position in the genome? Similarly, there is little interpretation of the actual values of phi. But there are many interesting questions, such as whether the long contexts learned by the model are indicative of sequence contexts that have a mutagenic effect (hotspots) or a protective effect. The manuscript would be improved*

**Author Response: We thank the reviewer for these comments (although note some slight abridgement here at the end!) and agree the paper would be improved with more context around our parameter estimates to tie them back to biology. In the revision, we have added some exploratory analyses to build this out a bit further and provide the reader with a more intuitive understanding of what these models tell us about the context dependence of mutation probabilities. Motivated by the reviewer’s suggestions, we investigated the following:**

a. **The volatility of sequence context dependence across window size in 9-mers (Supplementary Fig. 4A). For the average 9-mer context, about 2-3 edges are estimated to significantly affect the ultimate polymorphism probability. Very few 9-mer contexts (~10,000) were unaffected by any nucleotide expansions.**
b. **The largest sequence context size that affects polymorphism probabilities in 9-mers (Supplementary Fig. 4B). Approximately 60% of 9-mer sequence contexts have the entirety of their variability captured in 5-mer and smaller sequence context windows.**
c. **The magnitude of phi parameters across sequence context window sizes (Supplementary Fig. 4C). First, for Baymer to discover large probability changes in edges at wider sequence context windows, phi generally needs to be larger to overcome the higher general sparsity. Secondly, since smaller contexts are nested within more wider contexts that actually exist in the genome, large phis in these edges would be more impactful overall, which means there is presumably a higher degree of constraint on such contexts. Both of these hypotheses are consistent with the trend of greater phi values in larger context windows.**
d. **How much variability there is in different mutation types that is captured by edges between 3-mer and 9-mer nodes (Supplementary Fig. 4D). Notably 9-mer CpG>T context mutations are more similar to their nested 3-mer sequence contexts than any other mutation type, suggesting the smallest degree of variability in these edges, measured not only in total quantity (Fig 3F) but also in magnitude.**

**Please see our added descriptions on lines 257-271 of the Main Text.**

**In addition, we have also included analysis to infer rates beyond humans using extant polymorphism data from non-human, great ape species.**

Minor comments

*General, but for instance Figure 2D 2E 2F y axis labels. There is a proliferation of different terminology around the parameters of the model that I found confusing. The phi are sometimes referred to as "shifts", but then also can be referred to as "edges" (as in the Figure 2 caption) or sometimes it seems are called contexts (e.g. line 214 "We note that over 61% of all sequence contexts with PIP>.95 [...]"). And then inclusion means I=1? Or can it include I=0 but phi \neq 0?. And then differences in polymorphism rates between populations are not shifts in polymorphism rates but rather "shift differences". I suggest simplifying the terminology here to the extent that is possible.*

**Author Response: We apologize to the reviewer for any confusion caused. We have revised the manuscript to stick with a consistent use of terminology, i.e., “edges” whenever possible.**

*Line 255 I was confused about the exact criteria for inclusion in Table 1.*

**Author Response: We apologies for the confusion on this point.**

**We opted to include contexts that fulfilled two criteria:**

**(1) The probability that the polymorphism probability is the same is less than 0.01 (ie., the fraction overlap of estimated polymorphism probabilities < 0.01) and**

**(2) that the edge directly leading to the context also had a probability of being the same less than 0.01 (ie., the fraction overlap of estimated edge parameter directly preceding context node < 0.01).**

**This set of criteria was motivated by trying to identify inflection points in the tree. For example, while any context that contains TCC in its nested 3-mer context will have a different estimated polymorphism probability for mutations to thymine between European and African datasets, the true inflection point is most likely localized at the 3-mer level. These were the signals we were trying to capture. We mention the criteria in lines 308-321 and have edited the manuscript to be more explicit.**

*Line 527 Is y defined?*

**Author Response: We thank the reviewer for catch this. We have now defined *y* in the manuscript. (S1 Text line 52)**

*Line 553 Does "informative" mean I=1?*

**Author Response: We again apologize for the confusion on this point. Here, we mean that the polymorphism probabilities are identifiable, such that the mutation probabilities estimated for each intermediate node are also representative of that context’s mutation probabilities. Without this constraint, there are no guarantees that intermediate nodes would have estimated polymorphism probability estimates that correspond to their “true” values. In such a model only the leaf nodes would have accurate estimates. In the revised manuscript, we have replaced “informative” with “identifiable” (Main text line 165 and S1 text line 77) to hopefully mitigate this confusion.**

*Line 577 The level-by-level sampling scheme was quite unclear to me. Since there are multiple samples of the parameters at each level, how do these multiple samples play into the sampling at subsequent levels?*

**Author Response: Each layer is sampled independently in order, building up the tree. The distributions for each edge, estimated after each layer, are sampled from it to inform estimates for the next layer of the tree. We have added this step explicitly to the algorithm description to make it more clear (S1 text line 95-96).**

\# Reviewer 3

*The manuscript introduces a new method called Baymer that can estimate mutation rates based on sequence context. It builds a hierarchical model of the sequence context and solves the model selection problem of choosing the best context size taking the data sparsity into account by using a regularizing “spike and slab” prior. The authors first use the model to investigate differences in the sequence context of polymorphisms between populations. Secondly, they explore using polymorphisms to predict the rate of de novo mutations.*

*A few other recent articles have looked at methods for building 9mer models of mutations, but this is a new approach to doing so. I like the idea of the grafted tree "transfer learning" model where de novo mutations are used to train the upper levels in the hierarchical model, but polymorphism data is used to train the differences between the longer k-mers. Overall the manuscript is well-written and interesting.*

**Author Response: We thank the reviewer for their careful reading and their appreciation of the methodological aspects and interest in the work.**

*You write that a limitation of the model is the treatment of multi-allelic sites because you assume that you only see one mutation at each site. I think that not considering recurrent mutations is a bigger problem than multi-allelic variants when you train on the polymorphism data. In a large population sample like gnomAD the majority of all possible CpG mutations are polymorphic, and because of the high mutation rate, there will have been recurrent mutations a many CpG sites. In line 340, you write that you were surprised that models trained on singletons or doubletons did not do better. I do not think this is so surprising - if you do not take the low number of CpG sites that are not already polymorphic into account you would significantly underestimate their mutation rate using the singletons. Was the model fit with singletons especially bad for CpG transitions? Either increasing the mutation counts to account for recurrent mutations or reducing the background context count to reflect that you cannot observe a singleton at an already polymorphic site should improve the models.*

**Author Response: We thank the reviewer for this astute point. In the revised manuscript, for the singleton and other models, we have calculated likelihoods excluding/only including CpG contexts, which shows a marked improvement in performance (Supplementary Table 4), lines 413-417). These data suggest that deficiencies in singleton models are due to population matching, quality of variant calls, and recurrent mutations.**

*I tried installing the software using the included install script, but it failed because conda could not find many of the specific package versions specified in the yaml file. Maybe you need to specify which conda channels should be used?*

*I was able to get it installed by using other versions of the packages than the ones specified in the conda yaml file, and I tried running the code in “baymer_tutorial.ipynb”. But It looks like the tutorial in the notebook is not up to date with the names of the parameters in the functions it calls. When I tried counting mutations by running the second cell in the notebook, it gave me this error:*

*“TypeError: driver() missing 1 required positional argument: ‘quality_bool’”*

*In the 4th cell I got this:*

*TypeError: driver() got an unexpected keyword argument ‘random_seed’*

*In the 5th cell:*

*TypeError: driver() got an unexpected keyword argument ‘param_config_file’*

**Author Response: We sincerely apologize for this error! Edits were made to certain pieces of the code-base since submission (working on another project), and we neglected to update the tutorial. Having easily usable, and clean, code is very important to us. We have now updated the github to a cleaner state.**

*Can you say something about what the running time of the method is and whether it could be used on 11mers?*

**Author Response: There are no structural limitations of expansions beyond 9-mers, beyond computational time. We have added a section on computational time estimates to the manuscript, given our compute infrastructure (S2 Text).**

\# Reviewer 4

*The authors present a new model and computational implementation called Baymer for inferring polymorphism probability (a proxy for mutation rate) from nucleotide sequence context. Whereas most previous work has focused on fixed local sequence context (usually 3-mers), the key insight of Baymer is that related mutation contexts (e.g. two 5-mers with a common central 3-mer) may be expected to have similar polymorphism probabilities. They construct a hierarchical tree of increasing context size around a root singlet mutation type, and model the log probability of polymorphism as additive over parameters associated to tree edges. This parameter sharing of related contexts allows more reliable estimation, especially for larger contexts that are sampled very sparsely. They also introduce explicit sparsity assumptions on the edge weights via a spike-slab prior. This parameterization and regularization results in a compressed representation of polymorphism probability over a range of context sizes, and is more robust than empirical counts in terms of several out-of-sample prediction tasks.*

*Overall, I found the methods and results to be compelling, creative, rigorously validated, and mostly described well. Some aspects are somewhat heuristic and less interpretable, but overall I think the work shows that the model is behaving sensibly and improving on simpler baselines, although there are some directions for improvement.*

**Author Response: We are very grateful and appreciate the reviewer’s positive sentiments, considering the strengths and weaknesses of the implementation.**

*Major comments:*

*As the authors note, the alternating addition of 5’/3’ nucleotides across tree levels is somewhat awkward and not directly interpretable. It should also be noted that this excludes half of all even-ordered contexts. For example, as shown in figure 1, level 2 captures dinucleotide mutation types like A*>C* by adding nodes for the 4 possible 3’ contexts of the focal site. However, IIUC there are no nodes in any tree level that correspond to dinucleotide mutation with 5’ context, e.g. *A>*C. This asymmetry seems to omit a large number of mutation types from consideration, and also seems inconsistent with the approach of collapsing variants by reverse complement (with all focal site ancestral states collapsed to A or C). Are results robust to swapping the alternation order?*

**Author Response: We fully appreciate this comment, and Reviewer #2 points out a similar concern. In the revision, we now directly investigate this point.**

**To formally demonstrate the value of considering asymmetric steps, we hypothesized that these ‘even’ (asymmetric) steps, regardless, would generate more likely, more parsimonious models compared to only “odd” (symmetric) step model incorporation (Figure 2A). In the revised manuscript, we include new analysis to demonstrate that:**

a. **Selecting either “left” or “right” directions generate strongly correlated rates that are ultimately emitted from the model, indicating that the rates ultimately emitted from either choice of direction are robustly inferred (Figure 2B, 2C).**
b. **That the number of parameters utilized by the model is substantially less for those “asymmetric” even-window models, compare to the symmetric, odd-window sizes (Figure 2D).**
c. **That the likelihood of the models generated by including even-sized windows with “Left” steps or even-sized windows with “Right” steps are equivalent – or in the case of 9-mers, more likely – than an alternative that only takes “symmetric” odd-sized windows (Figure 2E).**

**Taken together, despite the limitations of their formulation, the models themselves are demonstrably better fit to the observed data.**

*As a side remark related to the above point, I wonder if it’s possible to augment the tree with nodes for both 5’ and 3’ additions, and sum over paths (the tree would become a DAG). For example, TCC>T would be the child of both TC>T and CC>T, and the probability contributions could be additive over such parallel paths.*

**Author Response: Because the “odd” (symmetric) contexts end up in largely the same place (see our examination of the alternation pattern, Figure 2), we posit that any gains would be marginal, where the sparsity limits are reached, for deriving polymorphism probabilities. However, we agree that such a model could help better localize inflection points in the tree for our between-population analyses. This is an important direction for future study.**

*One notable limitation in the Baymer approach is that it doesn’t allow parameter sharing among mutation types that have the same ancestral state and context, but different derived states. Indeed, a separate parameter tree is constructed for each singlet mutation type. This may limit the ability to detect signatures of mutability for highly specific ancestral contexts with less specified derived states (i.e. parameter sharing for mutation targeting to identical/similar contexts, but different derived states).*

**Author Response: We agree with the reviewer and have added this point to our discussion as a limitation (line 586-590). Originally, we conceived of a single tree that would capture both context-specific and mutation-type specific variability, but the higher dimensionality of that model formulation led us to narrow the scope in the interests of computational efficiency. Currently, this information can only be inferred with post-hoc analysis, but think it could be a valuable future extension to the model.**

*Polymorphism probability is being interpreted as a proxy for mutation rate. To test this, it would be very interesting to perform a simulation study using a population genetic simulator (like msprime or slim), where the mutation rate can be parameterized according to the tree-based parameters and a standard demographic model. Even just asking how well the polymorphism-based estimates reflect the underlying mutation trees would be interesting, let alone additional modeling work to put the trees into a history inference procedure. As the authors note in the discussion, this is left for future work, so not a necessary addition for this work. But it may be straightforward to assess at some point how good a proxy for mutation rate is the polymorphism probability.*

**Author Response: We thank the reviewer and agree with the comments that this is a good avenue for future studies. Unfortunately, to the best of our knowledge, SLiM only currently allows for 3-mer models, meaning there is a gap in simulation infrastructure for considering expanded context models. We are however, very interested in the extent to which our uncertainty estimates capture the variation we would expect using simulation models as suggested.**

Minor comments

*Instead of the even-odd basepair test-train split, I would have thought windowed region (or whole chromosome) holdouts would be a natural way to hold out SNPs for testing. Did the authors consider this?*

**Author Response: In previous work, we utilize chromosome-based ‘hold-outs’, and generally found compatible results. We became aware of some evidence to suggest chromosomal differences in mutation rates possibly due to differing genomic compartments (doi.org/10.1093/oxfordjournals.molbev.a003744), and thought an even-odd base pair approach would randomize to address any of these potential effects.**

*I didn’t really follow the arguments about a possible 2nd signal in the TCC>T signature with a distinct CC>T subcomponent. Given the asymmetric leveling, isn’t it impossible in this model to have found the 5’ dinucleotide subcomponent TC>T?*

**Author Response: The reviewer is correct and we recognize the confusion given the absence of the TC>T subcomponent in our analysis. We have decided to remove this hypothesis from the manuscript.**

*It is not clear if SNPs and mutation context are polarized with respect to an inferred human ancestral state, or taken simply as REF/ALT states.*

**Author Response: We apologies for the confusion. SNPs were polarized with respect to an inferred human ancestral state (S1 Text lines 10-13).**

*The mutation types *AC>*CC was previously shown to be enriched in the 1KG Japanese population (Harris and Pritchard), but this was later found to be a likely cell line artifact (Anderson-Trocmé et al.). I did not see this artifactual signature in Baymer results—can you comment on why?*

**Author Response: We believe that this is because we utilized the New York Genome Center resequencing data, rather than the original 1KG dataset, which had the cell line artifacts described. Relatedly, this is why we decided to repeat the similar experiment performed in Aikens et al. 2019, as their results are confounded by these artifacts. We have made more explicit note of this in the manuscript (Line 324-327).**

*It is not clear how equation 1 is constrained to the unit interval (i.e. how it represents a probability). Given the priors on the phi parameters, apparently there is a nonzero probability that p will exceed unity. Similarly, elements of the p vector in equation 2 could exceed unity. How is this being handled?*

**Author Response: We apologize for omitting these constraints in the manuscript equation and have added them. We thank the reviewer for this note. In practice, samples are rejected that would result in undefined probabilities.**

*It’s great that the authors have made their code repository available on GitHub. Unfortunately, the install procedure failed on both linux and mac for me. I was able to install after editing the conda environment file heavily (removing many packages, removing all the version specifications, and removing the user-specific prefix). The tutorial notebook then failed due to a API usage error in `baymer.mutation_counter.driver()`. I suggest the authors revise the install procedure to be replicable across machines/users with minimal dependencies, and debug the tutorial notebook.*

**Author Response: We sincerely apologize for this error! Edits were made to certain pieces of the code-base since submission (working on another project), and we neglected to update the tutorial. Having easily usable, and clean, code is very important to us. We have now updated the github to a cleaner state.**
